# Lymphoplasmapheresis for Steroid‐Refractory Neuromyelitis Optica Spectrum Disorder: A Real‐World Multicenter Study in China

**DOI:** 10.1111/cns.70628

**Published:** 2025-10-10

**Authors:** Qiuming Zeng, Haobing Cai, Xi Yuan, Fei Jiang, Hongliang Li, Xiaohua Dong, Si Chen, Jing Li, Weiwei Duan, Song Ouyang, Weifan Yin, Haipeng Li, Yi Zhong, Jun Yin, Hongyu Long, Bijuan Li, Huan Yang

**Affiliations:** ^1^ Department of Neurology Xiangya Hospital, Central South University Changsha Hunan People's Republic of China; ^2^ Clinical Research Center for Neuroimmune and Neuromuscular Disorders Xiangya Hospital, Central South University Changsha Hunan People's Republic of China; ^3^ National Clinical Research Center for Geriatric Disorders Xiangya Hospital, Central South University Changsha Hunan People's Republic of China; ^4^ Department of Blood Transfusion Xiangya Hospital, Central South University Changsha Hunan People's Republic of China; ^5^ Department of Acupuncture and Tuina Rehabilitation The First Hospital of Hunan University of Chinese Medicine Changsha Hunan People's Republic of China; ^6^ Department of Neurology Rehabilitation Hospital of Hunan Province Changsha Hunan People's Republic of China; ^7^ Department of Neurology The Affiliated Changsha Hospital of Xiangya School of Medicine, Central South University Changsha Hunan People's Republic of China; ^8^ The “Double‐First Class” Application Characteristic Discipline of Hunan Province (Clinical Medicine) Changsha Medical University Changsha Hunan People's Republic of China; ^9^ Department of Neurology the Second Xiangya Hospital, Central South University Changsha Hunan People's Republic of China; ^10^ Department of Neurology The First People's Hospital of Chenzhou City Chenzhou Hunan People's Republic of China; ^11^ Department of Neurology Changde First People's Hospital Changde Hunan People's Republic of China

**Keywords:** AQP4 antibody, cytokine, lymphoplasmapheresis, neuromyelitis optica spectrum disorder, platelet

## Abstract

**Background:**

Lymphoplasmapheresis (LPE), an innovative hemopurification technique that integrates conventional plasma exchange (PE) with lymphopheresis, has demonstrated efficacy in various neuroimmune disorders by removing excessive or dysfunctional lymphocytes and pathological components from plasma. This study aimed to unveil the mechanisms of LPE and provide additional clinical evidence supporting its use in the acute treatment of steroid‐refractory NMOSD.

**Methods:**

This study included 105 acute‐stage steroid‐refractory NMOSD patients. The efficacy and potential mechanisms of LPE were clarified by monitoring severity scores, clinical laboratory parameters, immune cell subsets, and cytokine levels.

**Results:**

In the retrospective cohort, LPE proved effective in treating acute steroid‐refractory NMOSD and was non‐inferior to PE. In the prospective cohort, LPE significantly decreased Expanded Disability Status Scale (EDSS) and Activities of Daily Living (ADL) scores (*p* < 0.05). In both cohorts, erythrocytes, hemoglobin, and platelets decreased. Notably, LPE significantly reduced the proportions of activated platelet‐adherent monocytes, plasmablasts, and memory B cells, while increasing that of naive CD8^+^ T cell subsets (*p* < 0.001). Additionally, LPE significantly reduced the levels of aquaporin‐4 antibody (AQP4‐Ab), fibrinogen, erythrocyte sedimentation rate, C‐reactive protein, complements, immunoglobulin, and proinflammatory cytokines (*p* < 0.05).

**Conclusion:**

While further validation is warranted, our findings suggest that LPE could represent a potential acute‐phase treatment strategy for patients with AQP4‐Ab^+^ NMOSD, especially in those who are refractory to corticosteroid therapy.

## Graphical abstract

Lymphoplasmapheresis (LPE) may potentially represent a promising therapeutic approach for AQP4‐Ab^+^ NMOSD patients, particularly those with an inadequate response to glucocorticoid therapy. LPE appears to exert a comprehensive effect by modulating immune cell subsets, reducing inflammation, and lowering antibody titers.

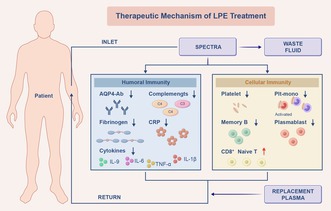

## 1 Introduction

Neuromyelitis optica spectrum disorder (NMOSD) is a rare autoimmune disease of the central nervous system (CNS) characterized by antibody‐mediated pathology [1, 2]. The disability of NMOSD patients primarily stems from acute phase injuries and rarely exhibits cumulative progression. Thus, early initiation of effective treatment is crucial for reducing disability and preventing recurrence.

Plasma exchange (PE) is a standard therapy for corticosteroid‐refractory or severe NMOSD, effectively reducing pathogenic autoantibodies [3, 4]. However, its mechanism is inherently limited as it cannot remove pathogenic cellular components like sensitized lymphocytes. Furthermore, the abrupt antibody depletion disrupts feedback inhibition, triggering compensatory antibody rebound that risks disease recurrence/exacerbation and necessitates repeated procedures [5]. This imposes significant burdens on patients, particularly the high plasma consumption—a critical challenge in resource‐constrained settings like China, where blood supply shortages impede healthcare development [6]. Consequently, there is an urgent need for therapies equally effective against steroid‐refractory NMOSD but designed to mitigate plasma resource constraints.

Lymphoplasmapheresis (LPE) represents a novel hemopurification approach that integrates conventional PE with lymphocyte apheresis, which aims to eliminate excessive or dysfunctional blood cells [7]. This comprehensive approach significantly enhances efficacy, shortens the course of the disease, and significantly reduces the consumption of plasma. Our and other teams' prior studies have verified LPE's efficacy in myasthenia gravis, Guillain‐Barré syndrome, and other autoimmune diseases [8, 9, 10]. Nevertheless, the efficacy of LPE in NMOSD has not been fully established. For acute NMOSD patients in China, the current treatment protocol involves initial glucocorticoid pulse therapy, with PE or immunoglobulin treatment only considered if ineffective. Under such NMOSD treatment circumstances in China, we initially probed into the efficacy and potential mechanisms of LPE in glucocorticoid‐refractory NMOSD patients during the acute phase.

In this study, we conducted a real‐world study building upon our prior LPE research [5, 7, 8, 9, 10]. In the retrospective cohort, we compared the efficacy of LPE with conventional PE in the treatment of acute attacks of steroid‐resistant NMOSD. In the prospective cohort, we validated the efficacy of LPE and elucidated the potential therapeutic mechanisms of LPE. Combining retrospective and prospective cohorts, our real‐world study sought to provide additional clinical evidence supporting its use in the acute treatment of NMOSD.

## 2 Materials and Methods

### 2.1 Study Design

**FIGURE 1:**
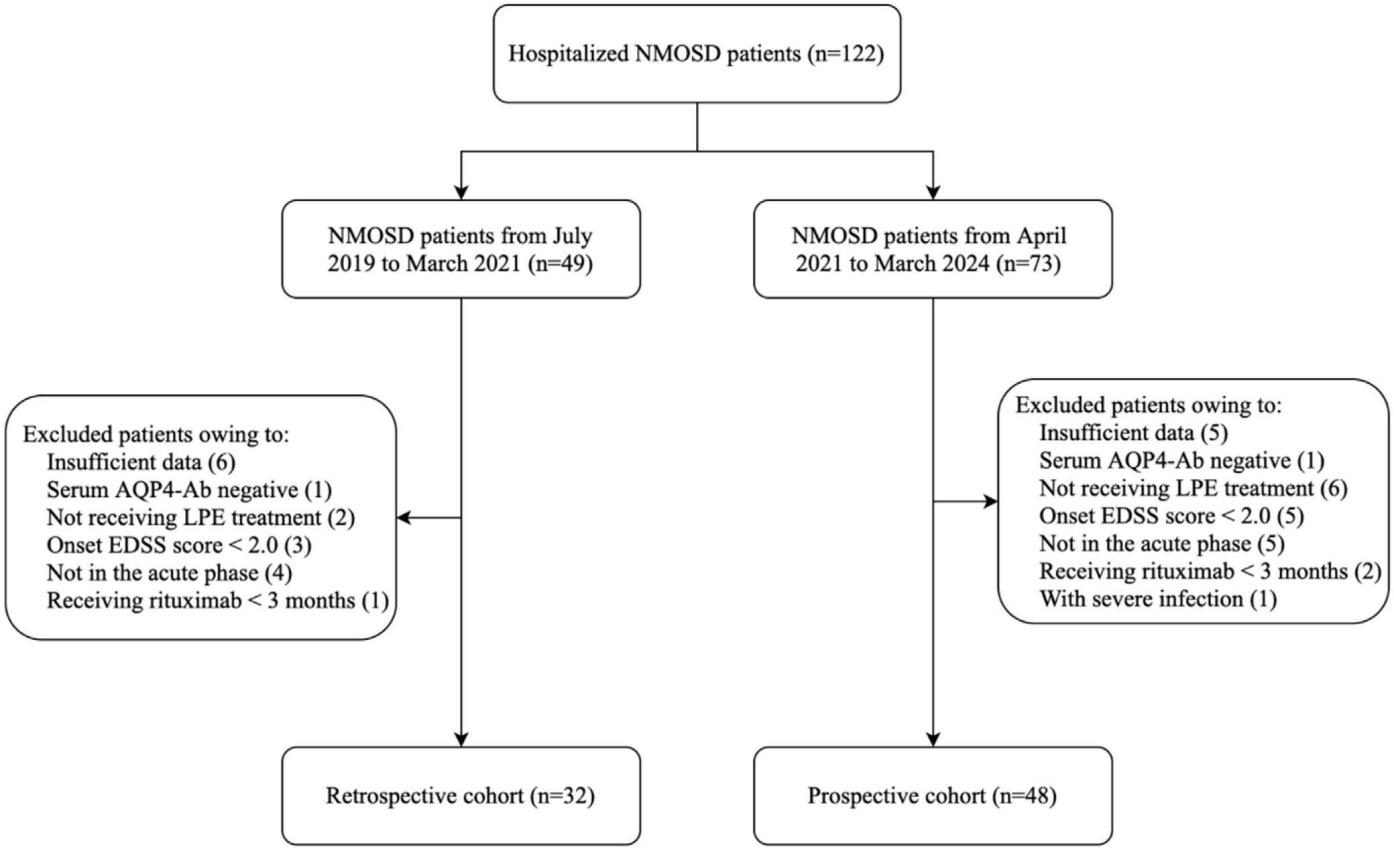
Study flow chart.

All patients were recruited from multiple centers in our study, including Xiangya Hospital, the Second Xiangya Hospital, the affiliated Changsha Hospital of Xiangya School of Medicine, Changde First People's Hospital and the First People's Hospital of Chenzhou City. In the retrospective cohort, a total of 32 NMOSD patients (29 females and 3 males) who underwent LPE from July 2019 to March 2021 and 25 NMOSD patients (22 females and 3 males) who underwent conventional PE during the same period were included. The prospective cohort comprised 48 NMOSD patients (42 females and 6 males) who received LPE from April 2021 to March 2024 (refer to Figure 1 for specific details). Disease severity was evaluated using the Expanded Disability Status Scale (EDSS) and the Activities of Daily Living (ADL) scale. The inclusion criteria were defined as follows: (1) Participants meeting the 2015 International Panel for NMO Diagnosis (IPND) diagnostic criteria for NMOSD with positive serum AQP4‐Ab (cell‐based transfection immunofluorescence assay); (2) Symptoms persisting for at least 24 h within 30 days of the current onset, with more than 90 days having passed since the previous acute NMOSD episode; (3) EDSS score of ≥ 2.0 points at the time of onset. If there was a prior EDSS score in the stable period, an increase of ≥ 1.0 point was required when the EDSS score was ≤ 5.5 points before, or an increase of ≥ 0.5 point when the EDSS score was > 5.5 points before; (4) Participants received IVMP within 1 month of the onset but there was insufficient improvement in the EDSS score (< 0.5 point). (5) No use of biological therapy (such as rituximab, satralizumab, etc.) in the past 6 months. Exclusion criteria were defined as follows: (1) hemodynamically unstable patients; (2) individuals with severe infection; (3) History for allergy to human albumin or fresh frozen plasma (FFP); (4) Presence of severe mental disorders or other chronic diseases; (5) individuals with pregnancy. In the retrospective cohort, general clinical data and EDSS scores were gathered before and after LPE treatments. The blood count results were only gathered before and after LPE treatments. In the prospective cohort, general clinical data, EDSS and ADL scores were collected before and after two LPE treatments, while peripheral blood samples and clinical laboratory testing were performed within 24 h before and after each LPE treatment.

Before LPE treatment, patients without contraindications to glucocorticoids in both study cohorts had previously received methylprednisolone at a dose of 1000 mg/day, administered in a stepwise manner, maintaining each dose for 3 days until discontinuation. Subsequently, patients were sequentially treated with tapered oral steroids or immunosuppressive agents. Each NMOSD patient underwent two LPE treatments during hospitalization, with an interval of 3–4 days between sessions. LPE was performed using the COBE Spectra apheresis system (Terumo BCT) equipped with the white cell channel. Lymphoid cells were collected via density gradient centrifugation and optical density detection within a manual program. The autologous plasma return line was diverted to fill the collection bag. Lymphocytes and peripheral blood mononuclear cells (PBMCs) were removed, while FFP was infused through the heparin cap's proximal port. Patient red blood cells were then combined with the FFP, and the mixture was reinfused. Approximately 70%–80% of total blood volume (20–25 mL/kg) was exchanged per procedure, replaced with FFP or cryoprecipitate‐reduced plasma [7]. The control group underwent PE according to standard procedures [10] comprising three to five sessions. Sessions occurred every other day, with approximately 1–1.5 times the total blood volume (35–40 mL/kg) exchanged per procedure.

### 2.2 Immunofluorescence Assay to Detect Serum AQP4‐Antibody

Serum was isolated from 5 mL peripheral blood and stored at −80°C. Serum was equilibrated to room temperature and diluted according to a concentration gradient before testing. The slides contained cells with overexpressed antigen AQP4, which could bind AQP4‐Ab in the sample. Antibodies bound to the cells were recognized by FITC‐labeled anti‐human IgG secondary antibodies. Finally, the presence of autoantibody in the sample was judged according to the fluorescence signal. The titer of a positive sample is usually taken as the lowest concentration at which a positive result can be obtained in that serum.

### 2.3 Luminex Technology to Detect the Expression of Multiple Cytokines in Serum

Multiple cytokines in serum were detected by magnetic bead suspension array using the Bio‐Plex Pro Human Cytokine Screening Panel, 27‐plex (Bio‐Rad Laboratories, Hercules, CA, USA). The commercially available Human Cytokine 27‐plex Kit (Bio‐Plex Pro, #M500KCAF0Y) was used following the manufacturer's instructions. Results were analyzed using the Bio‐Plex Manager software, and the concentrations of cytokines were calculated from the standard curve.

### 2.4 Flow Cytometry to Detect the Proportion of Cell Subsets

Flow cytometry assay was performed according to the protocol published previously [11, 12, 13]. For platelet assay, whole blood samples were collected into the BD Vacutainer Citrate Tubes containing 0.106 (3.2%) mol/L trisodium citrate anticoagulant. For the detection of other cell subpopulations, whole blood samples were all collected into the BD Vacutainer K2E(EDTA) Tubes. Flow cytometry panels and antibodies used in this study were summarized in Table S1. Samples were run on a DxP Athena flow cytometer (Cytek Biosciences, USA), and the data files were analyzed by gating in the light‐scatter histogram on cells using Flowjo V10.8 software.

### 2.5 Statistical Analysis

Data conforming to normal distribution were expressed as mean ± standard deviation, and those not conforming to normal distribution were expressed as median and interquartile range. Frequency and percentage were used for categorical variables. The data conforming to normal distribution were compared by paired *t* test, and the data not conforming to normal distribution were compared by paired rank sum test. Mean or median imputation was used to handle the very limited missing data. *p* value < 0.05 was defined as statistically significant. SPSS 26.0 was used for statistical analysis, Graphpad Prism 9.4.1 and Adobe Illustrator 2022 were used for drawing and layout.

## 3 Results

### 3.1 Demographic and Clinical Characteristics

A total of 32 NMOSD patients treated with LPE and 25 NMOSD patients treated with PE were included in the retrospective cohort. The comparison of clinical features between the two groups is shown in Table S2. Meanwhile, in the prospective cohort, 48 NMOSD patients were enrolled, comprising 42 females and 6 males, with a mean age of 42.81 ± 15.38 years and a mean onset age of 39.27 ± 16.91 years. In both groups, the median interval between ending IVMP treatment and the first LPE is within 1 week. Also, other autoimmune disorders were present in over 30% of patients, and acute myelitis was the most prevalent initial symptom. No significant differences in clinical characteristics were observed between the two cohorts. Detailed patient characteristics are provided in Table 1.

**TABLE 1:** The patient characteristics of retrospective and prospective cohorts.

	Patients		*p* value
	Retrospective study (*n* = 32)	Prospective study (*n* = 48)	
Female sex (*n*, %)	29 (90.6%)	42 (87.5%)	> 0.05
Age (y; mean, SD)	46.91 (16.20)	42.81 (15.38)	> 0.05
Age at onset (y; mean, SD)	43.97 (16.17)	39.27 (16.91)	> 0.05
Course of disease (m; median, IQR)	15.50 (58.00)	14.00 (71.10)	> 0.05
ARR (n^a^; median, IQR)	0.62 (1.85)	0.38 (0.69)	> 0.05
Time from the end of IVMP to the first LPE (d; median, IQR)	6.00 (3.00)	5.00 (2.00)	> 0.05
Autoimmune disorders (*n*, %)			> 0.05
Autoimmune thyroid disorders	6 (18.8%)	8 (16.7%)	> 0.05
Sjögren's syndrome	5 (15.6%)	9 (18.8%)	> 0.05
Rheumatoid arthritis	0 (0)	1 (2.1%)	> 0.05
Use of Immunosuppressive agents (n, %)			> 0.05
Mycophenolate mofetil	11 (34.4%)	17 (35.4%)	> 0.05
Tacrolimus	2 (6.3%)	2 (4.2%)	> 0.05
Azathioprine	1 (3.1%)	0 (0)	> 0.05
Initial symptoms (*n*, %)			> 0.05
Optic neuritis	13 (40.6%)	21 (43.8%)	> 0.05
Acute myelitis	16 (50.0%)	25 (52.1%)	> 0.05
APS	1 (3.1%)	1 (2.1%)	> 0.05
Brainstem syndrome	2 (6.3%)	1 (2.1%)	> 0.05

Abbreviations: APS, area postrema syndrome; ARR, annualized relapse rate; IQR, interquartile range; SD, standard deviation.

^a^ Only patients with relapses.

### 3.2 LPE Efficacy and Complete Blood Count Changes in the Retrospective Cohort

**FIGURE 2:**
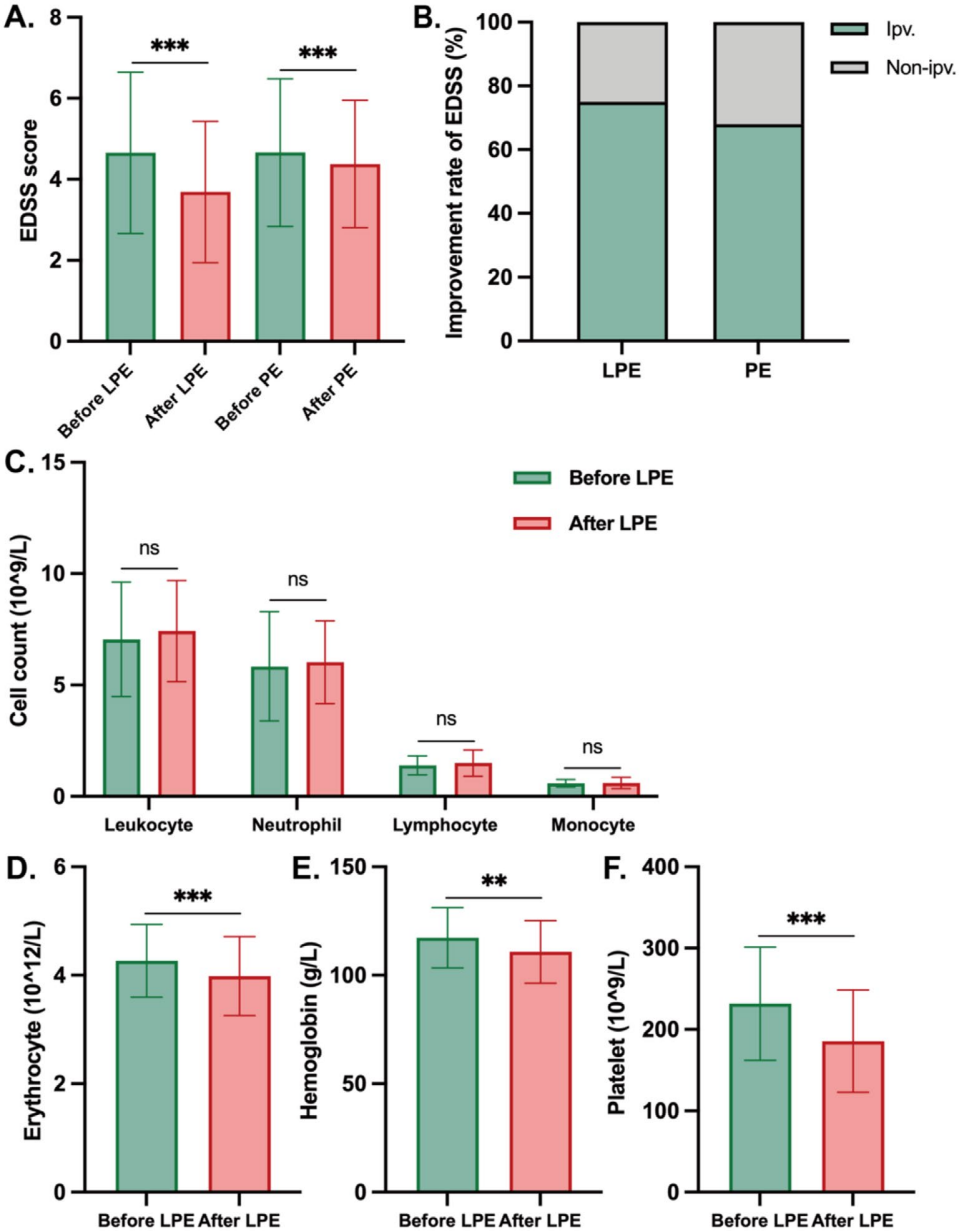
Changes of EDSS scores and blood counts in the retrospective cohort. (A) Changes of EDSS scores before and after LPE or PE. (B) Improvement rate of EDSS scores for LPE and PE in the retrospective cohort. (C–F). Changes of blood counts before and after two LPE treatments. Ipv, improvement; Non‐ipv, Non‐improvement; ns = no significance. *p < 0.05; **p < 0.01; ***p < 0.001.

In the retrospective cohort, the median EDSS score of 32 patients treated with LPE decreased from 4.5 to 4.0 following two LPE treatments (*p* < 0.001). Among 25 patients treated with PE undergoing three to five treatments, the median EDSS score also decreased from 4.5 to 4.0 (*p* < 0.001), albeit with a slightly lower EDSS improvement rate compared to LPE. This observation confirms that LPE is non‐inferior to PE in efficacy and requires fewer treatment sessions for patients. Additionally, complete blood counts were conducted, revealing a significant decrease in the abundance of erythrocytes, hemoglobin, and platelets after the two procedures in patients receiving LPE treatment (*p* < 0.05). However, there were no significant changes in the abundance of leukocytes, neutrophils, lymphocytes, and monocytes (*p* > 0.05) (Figure 2 and Table S3).

### 3.3 LPE Efficacy in the Prospective Cohort

**FIGURE 3:**
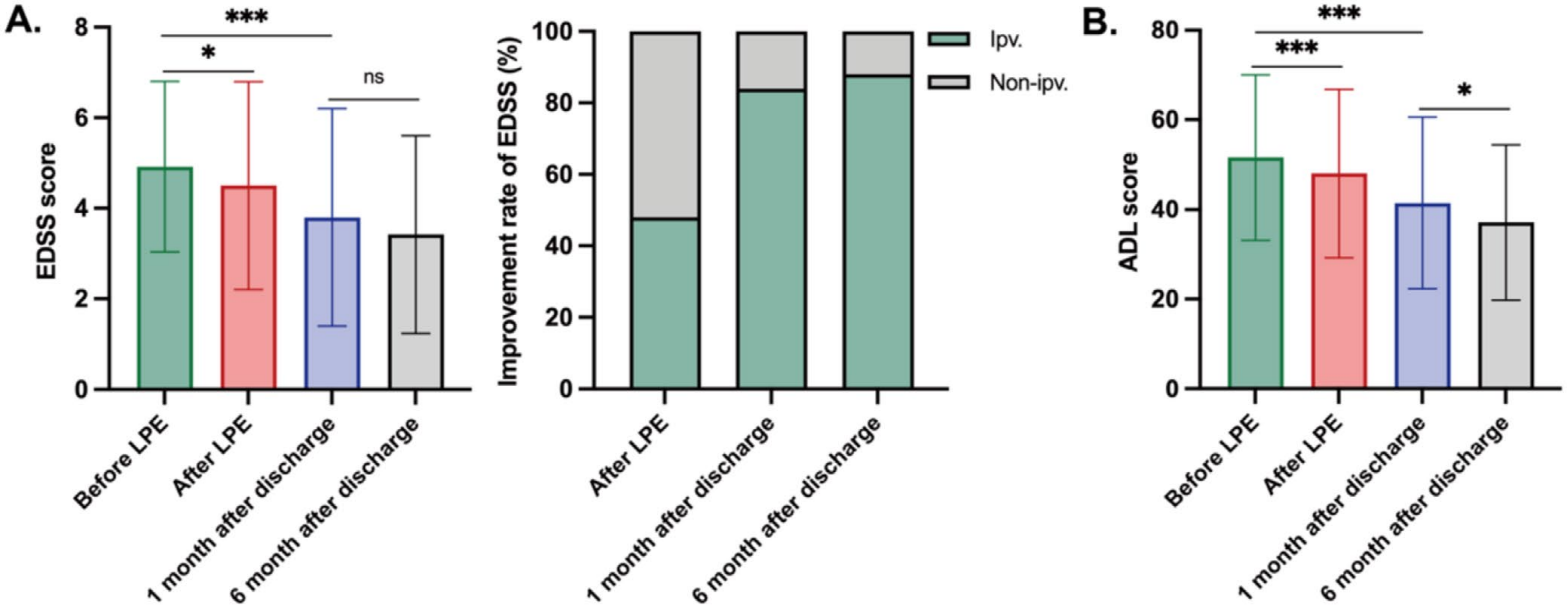
Changes of EDSS and ADL scores during the hospitalization and six‐month follow‐up in the prospective cohort. (A) Changes of EDSS scores and improvement rate during the hospitalization and 6‐month follow‐up. (B) Changes of ADL scores during the hospitalization and 6‐month follow‐up.

In the prospective cohort, the median EDSS score of the 48 NMOSD patients decreased from 4.50 to 4.00 following two LPE treatments. Notably, it further declined to 3.00 1 month after discharge, remaining stable at 3.00 at 6 months. The improvement rate of EDSS was 48% after two LPE treatments, 84% at 1 month after discharge, and 88% at 6 months after discharge. Concurrently, the median ADL score was 56.00 at admission, reducing to 48.00 after treatment, 38.00 at 1 month after discharge, and 35.00 at 6 months after discharge. These findings highlight the add‐on impact of LPE on enhancing disability and the clinical prognosis of NMOSD patients (Figure 3).

### 3.4 Complete Blood Count Changes in the Prospective Cohort

**FIGURE 4:**
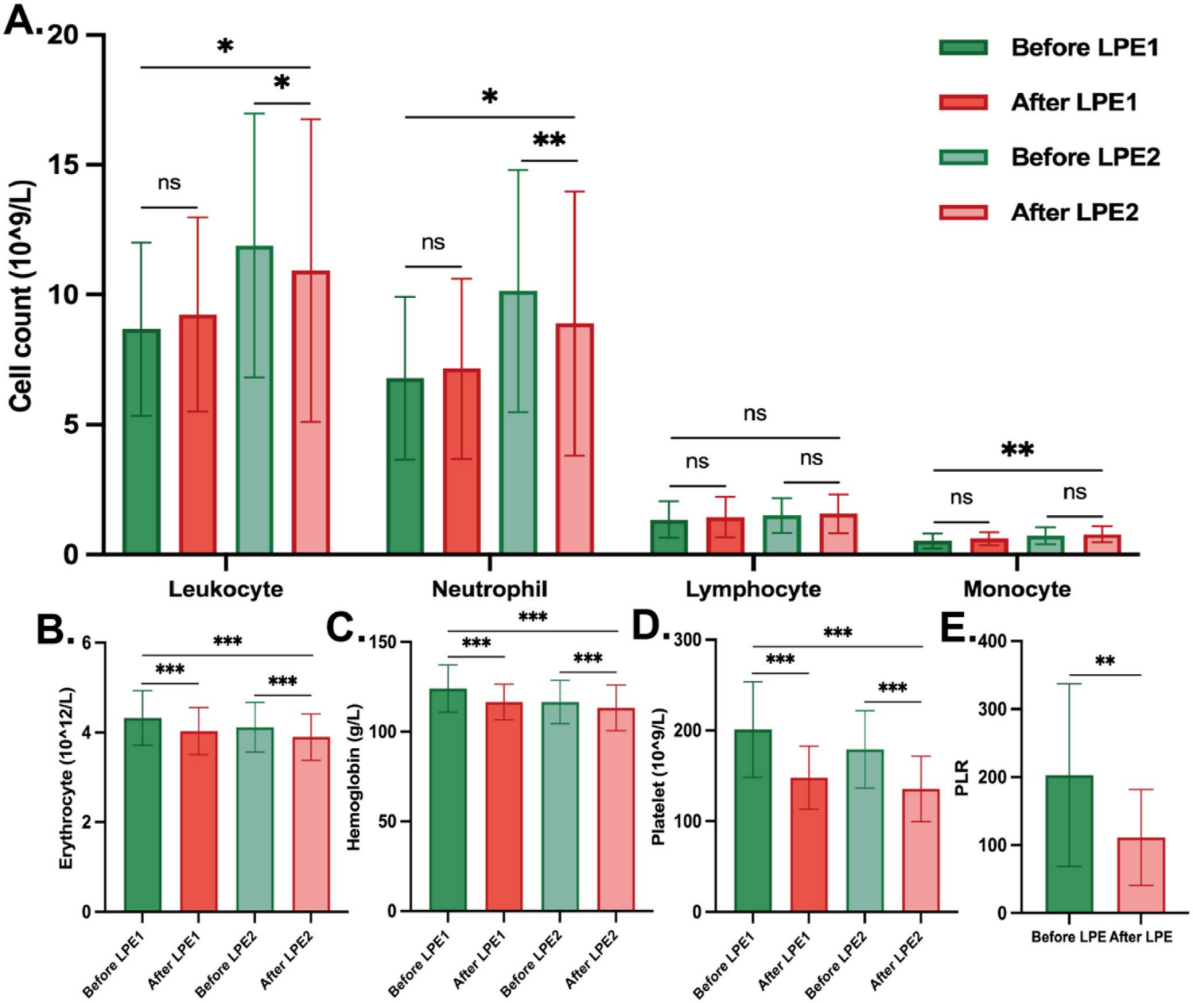
Changes of blood counts in the prospective cohort. (A–D) Changes of blood counts before and after two LPE treatments, respectively, in the prospective cohort. (E) PLR before and after two LPE treatments. PLR, platelet‐to‐lymphocyte ratio.

It was observed that the counts of leukocytes, neutrophils, and monocytes increased significantly after two LPE treatments compared with the baseline levels (*p* < 0.05). Notably, both leukocytes and neutrophils exhibited a rebound trend after the first LPE treatment, with the cell count before the second LPE treatment being significantly higher than that before the first LPE treatment (*p* < 0.05). Consequently, although the second LPE led to a decrease in leukocytes and neutrophils after a single LPE treatment (*p* < 0.05), the overall increase remained higher than the pre‐treatment levels. In contrast, the counts of erythrocytes, hemoglobin, and platelets after two LPE treatments were significantly lower than those before treatment (*p* < 0.001), with a notable reduction in platelet count after the first LPE treatment. The platelet‐to‐lymphocyte ratio (PLR) also exhibited a marked decrease after treatments. However, there was no significant difference in lymphocyte count after two LPE treatments (*p* > 0.05) (Figure 4 and Table S4).

### 3.5 Flow Cytometry Assay of Peripheral Blood in the Prospective Cohort

**FIGURE 5:**
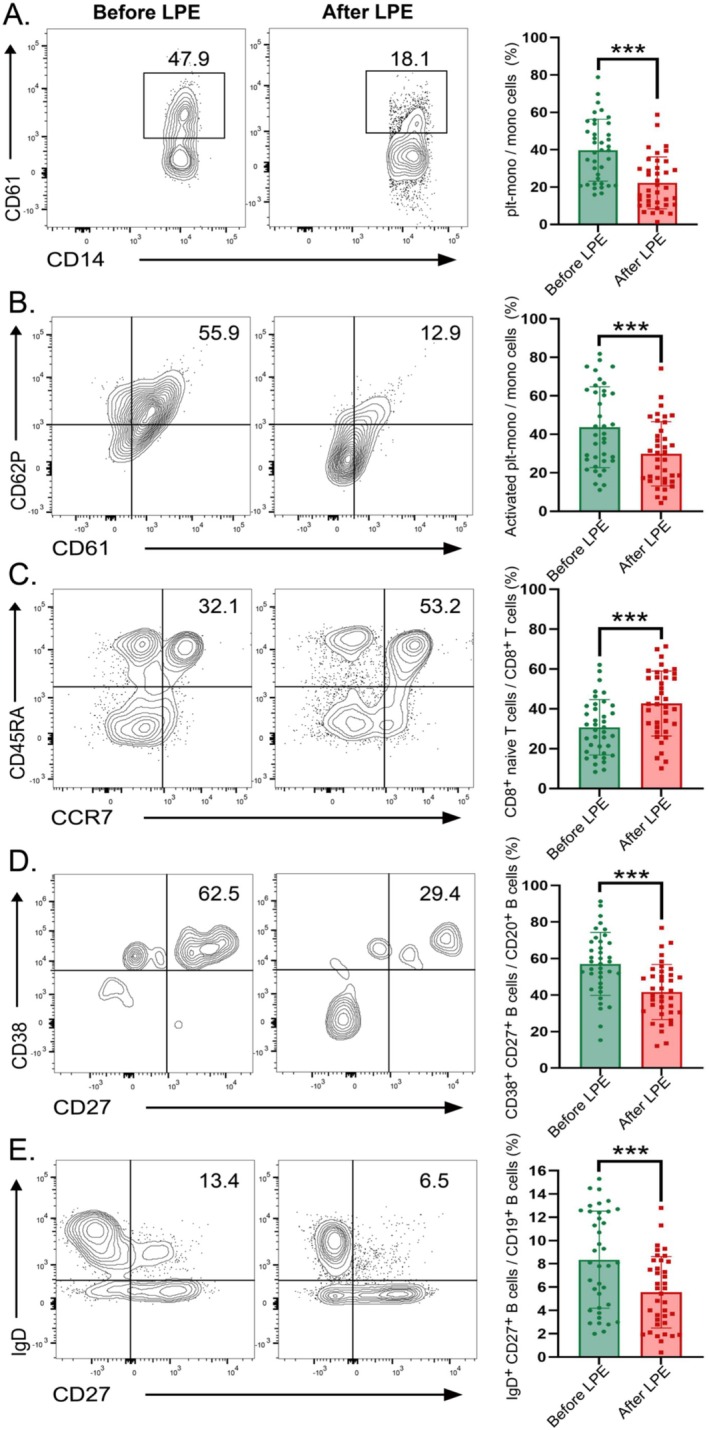
Peripheral blood flow cytometry profile in the prospective cohort. The ratio of platelet‐adherent monocytes (A), activated platelet‐adherent monocytes (B), naive CD8^+^ T cells (C), plasmablasts (D), and memory B cells (E) subsets before and after two LPE treatments.

Given that lymphocyte counts exhibited no significant changes before and after LPE treatment, we conducted a detailed analysis of immune cell subsets and platelets using flow cytometry in 38 patients before and after LPE treatments. The peripheral blood immune cell subsets and platelets analyzed included T cells (activated T, central memory T, naive T, effector T, effector memory T, Treg, memory Treg, activated Treg, naive Treg, Th1, Th2, Th17), B cells (naive B, memory B, plasmablast), NK cells, DC cells, monocytes, and platelet subsets. Following LPE treatment, there were significant reductions in the proportions of (activated) platelet‐adherent monocytes, plasmablasts, and memory B cells (*p* < 0.001). Simultaneously, the abundance of naive CD8^+^ T cell subsets increased significantly (*p* < 0.001) (Figure 5). However, there were no significant differences in the proportion of other cell subsets.

### 3.6 Changes in Inflammatory Parameters After LPE Treatment in the Prospective Cohort

**FIGURE 6:**
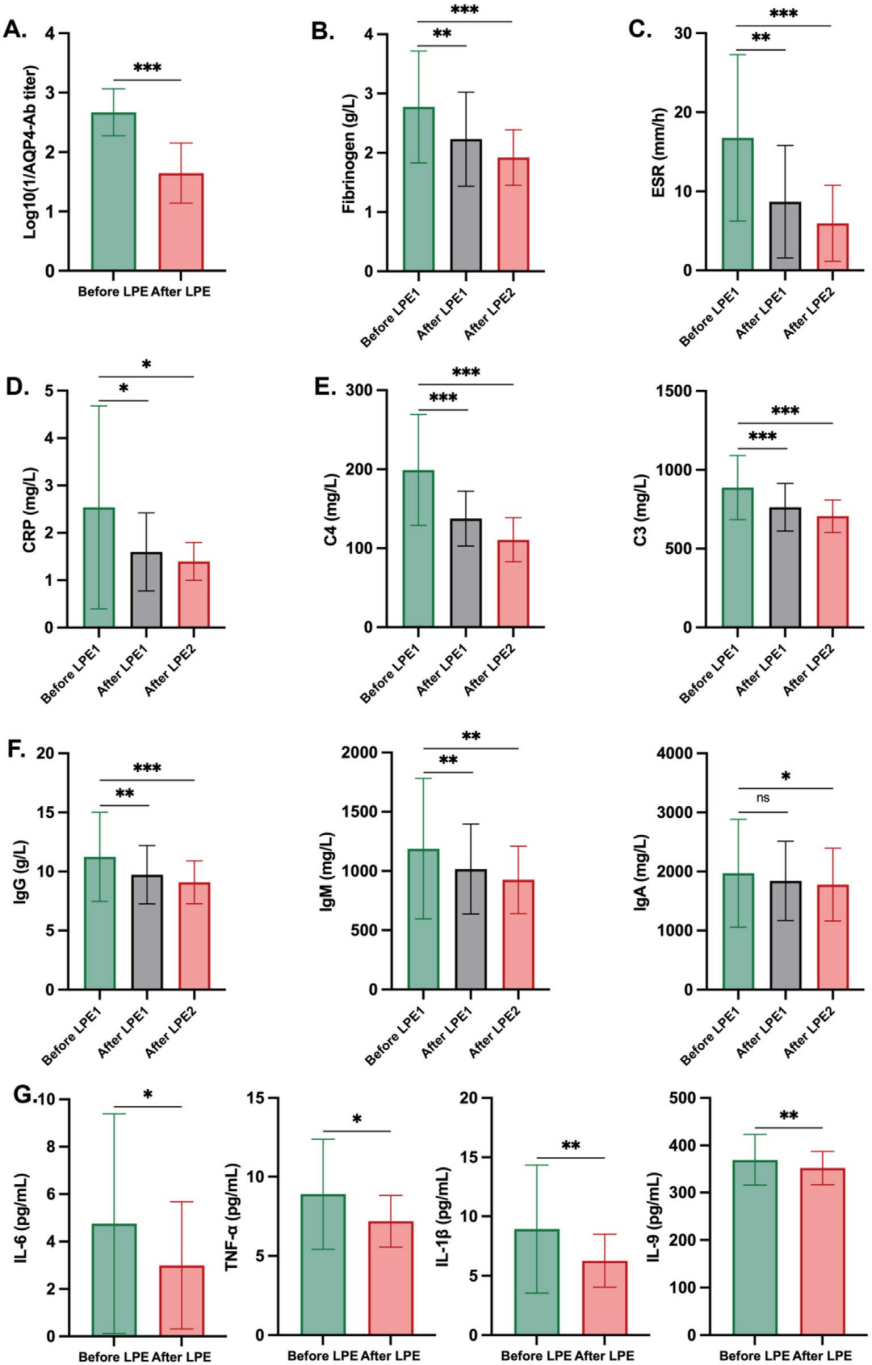
Changes of inflammatory mediators in the prospective cohort. (A–D) Changes of AQP4‐Ab titer, fibrinogen, ESR, CRP, and before and after two LPE treatments in the prospective cohort. (E) Changes of complements before and after LPE treatments. (F) Changes of immune globulins before and after LPE treatments. (G) Changes of cytokines before and after LPE treatments.

**FIGURE 7:**
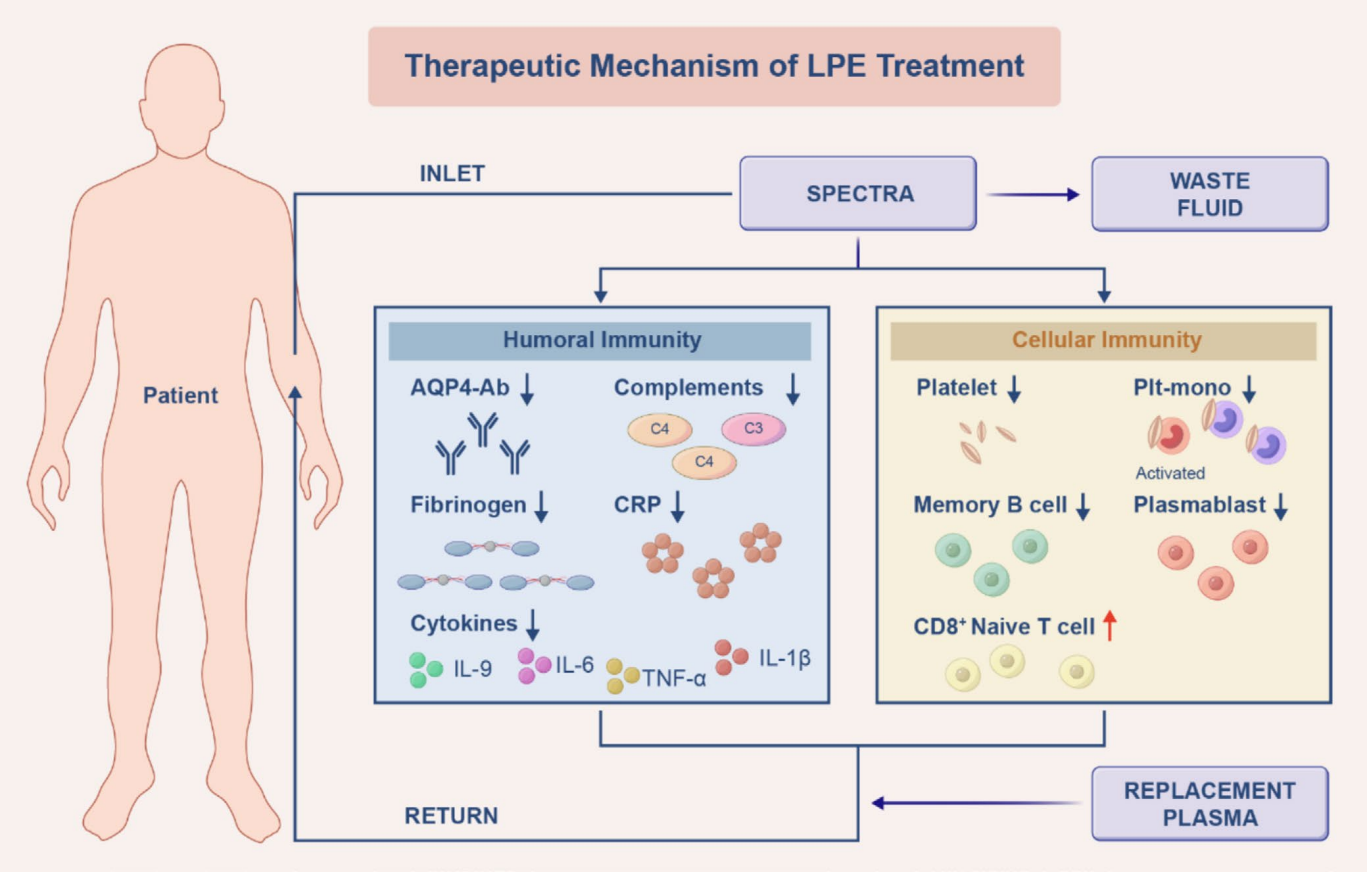
The therapeutic mechanism of LPE. Plt‐mono, platelet‐adherent monocytes.

To investigate whether LPE effectively reduces the levels of pathogenic antibodies, serum samples were collected from 48 patients after two LPE treatments for AQP4‐Ab titer detection. Following equivalent conversion, the median AQP4‐Ab level of patients before LPE treatment was 3.00, decreasing to 1.51 after treatment (*p* < 0.001), indicating the clearance of pathogenic antibodies by LPE. Moreover, other inflammatory mediators significantly decreased after treatment, including erythrocyte sedimentation rate (ESR), C‐reactive protein (CRP), fibrinogen, complement C4, complement C3, IgG, IgM, and IgA (Figure 6 and Table S4). By comparing the changes after two LPE treatments, it was observed that the efficacy of the first LPE treatment was higher than that of the second one. To comprehensively assess the effect of LPE on cytokines, a total of 27 cytokines in serum were detected using Luminex technology. The concentrations of four cytokines—interleukin‐6 (IL‐6), tumor necrosis factor‐α (TNF‐α), IL‐1β, and IL‐9—decreased significantly before and after LPE treatment. The detailed levels of the 27 serum multiplex cytokines before and after LPE treatment are displayed in Figure 6 and Tables S4 and S5. The comprehensive therapeutic mechanism of LPE is illustrated in Figure 7.

### 3.7 Adverse Effects of LPE

In both retrospective and prospective cohorts, 92.5% (74 of 80) patients were free of any adverse event. Only six mild adverse reactions were reported; five were allergic reactions (manifesting as rash and pruritus), alleviated by reducing the treatment flow rate and administering intravenous dexamethasone plus intramuscular diphenhydramine. One case of chills, associated with hypothermia, was prevented by warming the replacement fluid. None of the adverse reactions led to treatment discontinuation. Previous reports of common complications associated with PE, such as coagulopathy and hypotension, did not occur in our LPE treatment.

## 4 Discussion

Despite previous studies demonstrating that early IVMP in combination with PE can better enhance the prognosis of NMOSD patients, in China, due to the shortage of clinical blood supply and medical technology, NMOSD patients in primary medical institutions are more inclined to undergo IVMP first. In light of this reality, finding a more effective, less blood‐consuming, and more economical treatment approach is an urgent requirement for the acute treatment of NMOSD in China. LPE, as a modification of PE, which improves conventional PE by effectively eliminating pathological substances, can achieve the therapeutic goal with fewer treatment sessions and a reduced amount of blood consumption.

Nevertheless, due to the constraint of clinical blood usage, it was arduous for us to carry out a randomized controlled study to elucidate the therapeutic disparity between LPE and PE. Hence, only a retrospective comparative analysis between LPE and PE was performed, which confirms that LPE is non‐inferior to PE in efficacy and requires fewer treatment sessions for patients. A reduction in EDSS scores among NMOSD patients within the prospective cohort further substantiates the efficacy of LPE. Concurrently, ADL scores showed continuous improvement during the follow‐up, which suggested that LPE may have a positive effect on the recovery from acute attacks of steroid‐refractory NMOSD. Compared with PE, the lower incidence of adverse events in LPE is mainly attributed to the fewer treatment sessions required for LPE and the use of plasma substitutes in conventional PE.

To further explore the potential biological mechanisms of LPE, multiple laboratory tests were conducted before and after treatments. Blood sampling timing was based on pharmacokinetic principles to capture LPE's direct biological effects. Since LPE's core mechanism—rapid clearance of pathogenic molecules—occurs within hours, blood was collected within 24 h before and after LPE in this prospective cohort. This captured immediate effects on blood constituents, avoiding confounding from intravascular replenishment with delayed sampling. Given the presence of AQP4‐Ab in the blood is the main pathogenic factor of NMOSD [2], our prospective cohort study affirms that the AQP4‐Ab titer in NMOSD patients significantly decreases after two LPE treatments. Furthermore, LPE demonstrates efficacy in avoiding the antibody rebound associated with conventional PE by altering lymphocyte subsets.

The binding of IgG to AQP4 can lead to extensive deposits of complement activation markers (C9neo antigen) around fibrotic and hyalinized blood vessels. Multiple studies have demonstrated that levels of complement C3 are elevated in NMOSD patients compared to healthy controls. Moreover, C3 levels in these patients are correlated with disease activity, neurological dysfunction, and AQP4‐Ab positivity [14]. A Chinese study further confirmed that peripheral serum complement C3 is a significant influencing factor for severe motor disability (EDSS score ≥ 6 points), with peripheral complement C3 and C4 serving as predictors of severe motor dysfunction [15]. LPE can significantly reduce complement levels in NMOSD patients in this study, which benefits the recovery of neurological function in patients.

IL‐6 is a pivotal factor in promoting lesion formation in NMOSD by facilitating the infiltration of neutrophils or plasma cells. Its correlation with disease severity and EDSS scores has been established [16]. IL‐1β, released from NMOSD lesions, has been shown in an Austrian study to promote neutrophils, contributing to blood–brain barrier destruction alongside induced complements, laying a foundation for the rapid progression of NMOSD lesions [17]. Previous studies also indicated significantly higher serum TNF‐α levels in NMOSD patients compared to healthy controls [18]. IL‐9 has pleiotropic effects and participates in the pathogenesis of autoimmune diseases by regulating the functions of T/B cells [19]. Our study revealed that LPE could effectively reduce the levels of IL‐1β, TNF‐α, IL‐6, and IL‐9. This suggests that cytokine modulation may be one of the mechanisms through which LPE exerts its efficacy in NMOSD treatment.

Fibrinogen, the end product of the coagulation cascade, has been recognized as a regulator in the process of the inflammatory response [20]. Fibrinogen can induce macrophages to secrete proinflammatory cytokines [21]. A recent investigation found a significant correlation between fibrinogen levels and the severity of NMOSD, recurrence rate, and the number of spinal cord injury segments [22]. Our study observed a continuous decrease in the fibrinogen content of patients after two LPE treatments, which may contribute to the alleviation of the inflammatory response in the acute phase of NMOSD patients.

Both the generation of AQP4 antibodies and the elevation of pro‐inflammatory cytokines are closely associated with immune cells. In our retrospective cohort, the abundance of leukocytes, neutrophils, lymphocytes, and monocytes in 32 patients did not change significantly after two LPE treatments. However, in the prospective cohort, the blood collection timing for all enrolled patients was controlled within 24 h before and after each LPE treatment, revealing higher leukocyte, neutrophil, and monocyte counts in 48 patients after two LPE treatments compared to before. Among them, leukocytes and neutrophils rebounded significantly after the first LPE treatment. Therefore, even though leukocytes and neutrophils decreased after the second LPE treatment, the overall increase remained higher than that before the treatment. This rebound is mainly related to the apheresis of blood cells by LPE and is associated with prior glucocorticoid pulse treatment.

Besides, complete blood count may not accurately reflect changes in the immune status of NMOSD patients treated with LPE. We conducted a more detailed investigation into the changes of immune cell subsets following LPE treatment in NMOSD patients using flow cytometry. In the prospective cohort, LPE treatment was found to significantly decrease the proportion of (activated) platelet‐adherent monocytes, plasmablasts, and memory B cells in the peripheral blood, while the proportion of naive CD8^+^ T cell subsets increased. CD8^+^ T cells, as key players in neuroinflammation, participate in the immune cascade during the pathogenesis of NMOSD [23]. Plasmablasts are elevated in the active phase of NMOSD in AQP4‐Ab^+^ NMOSD patients [24]. Subsequently, plasma cells, when stimulated by IL‐6 (which is increased in NMOSD), can directly cause increased AQP4‐Ab secretion [25]. Memory B cells serve as key reservoirs for plasma cell generation in the secondary immune response [26]. One study showed that high‐dose glucocorticoid increased the proportion of memory B cells, while rituximab decreased it [27]. Our study confirmed a significant decrease in plasmablasts, memory B cells, and IL‐6 in the peripheral blood of NMOSD patients after LPE treatment. This reduction is beneficial for decreasing the secretion of pathogenic antibodies. This provides new insights into the potential therapeutic mechanisms of LPE.

Three types of blood components—erythrocytes, hemoglobin, and platelets—exhibited a similar decline after treatment in both the retrospective and prospective cohorts. This aligns with the findings of previous studies indicating that PE can reduce platelets [28]. Notably, in addition to regulating intravascular hemostasis, platelets also play a crucial role in innate immunity [29, 30]. Proteomic studies identified essential immune function‐related proteins among the molecules secreted by platelets, including IL‐1, major histocompatibility complex molecules, and CD40L [31, 32]. Through flow cytometry, we observed that LPE significantly reduced the proportion of platelet‐monocyte aggregation. Over the years, platelet count alterations have served as one of the observational indices for assessing NMOSD treatment efficacy in many clinical studies. A recent study suggested that PLR could predict the severity of neurological disability at 2 years in NMOSD patients [33]. In the prospective cohort, we confirmed a significant reduction in PLR after LPE treatments. Our study confirmed that LPE can reduce platelets, platelet‐monocyte aggregations, and PLR, suggesting that intervention in platelets may be an important part of the therapeutic mechanism of LPE.

In conclusion, while further validation is warranted, our findings suggest that LPE could represent a potential acute‐phase treatment strategy for patients with AQP4‐Ab^+^ NMOSD, especially in those who are refractory to corticosteroid therapy. LPE appears to exert a comprehensive effect by modulating immune cell subsets, reducing inflammation, and lowering antibody titers (Figure 7). Compared with most previous retrospective studies, our work represents one of the first explorations using a real‐world prospective cohort to examine the potential therapeutic mechanisms of LPE. We believe this approach provides additional insights to support future treatment strategies for AQP4‐Ab^+^ NMOSD during the acute phase. Additionally, LPE utilizes existing PE equipment, avoiding additional device needs while requiring only targeted staff training. By reducing plasma use and treatment sessions, it appears to offer lower per‐session costs than conventional PE. These features may support broader clinical implementation. Given the inherent limitations of real‐world studies, we plan to address these constraints in future research by conducting a registry‐embedded randomized controlled trial (RCT), expanding the sample size and follow‐up duration, enhancing geographical and demographic diversity, and performing comprehensive subgroup analyses to generate more robust evidence supporting the use of LPE as an acute‐phase treatment for NMOSD.

## Author Contributions

H.Y. and Q.Z. contributed to the study design. Q.Z. and H.C. performed data analysis and wrote the manuscript. H.C. performed patient screening, specimen collection, and data collection. F.J. performed and analyzed the flow cytometry data. X.Y., H.L., X.D., S.C., J.L., W.D., S.O., W.Y., H.L., Y.Z., J.Y., H.L., and B.L. participated in data collection. All authors read and approved the final manuscript and analysis.

## Ethics Statement

All procedures involving human participants were approved by the Medical Ethics Committee of Xiangya Hospital of Central South University (NO. 201904147‐2).

## Consent

Participants provided written informed consent prior to participating in the study.

## Conflicts of Interest

The authors declare no conflicts of interest.

## Supporting information


**Data S1:** Supporting Information.

## Data Availability

The data that support the findings of this study are available from the corresponding author upon reasonable request.
