# Supplementary material for: Lymphoplasmapheresis for Steroid‐Refractory Neuromyelitis Optica Spectrum Disorder: A Real‐World Multicenter Study in China
Source: CNS Neurosci Ther. 2025 Oct 10;31(10):e70628. doi: 10.1111/cns.70628 (PMC12513854; doi:10.1111/cns.70628)
Supplement: Supplementary file 1 — Data S1: Supporting Information. [file CNS-31-e70628-s001.docx]

**Lymphoplasmapheresis for steroid-refractory neuromyelitis optica spectrum disorder:**

**A real-world multicenter study in China**

**Supplementary materials**

**Table 1 Flow cytometry panels and antibodies**

| **Panels** | **Antibody** | **Conjugate** | **Source** | **Catalog no.** |
| --- | --- | --- | --- | --- |
| **Platelet** | GPVI | APC | BD Biosciences | 564701 |
|  | CD42b | PE | BD Biosciences | 555473 |
|  | CD61 | FITC | BD Biosciences | 557291 |
| **platelet-adherent monocytes** | CD11b | BV421 | BD Biosciences | 562632 |
|  | CD14 | PE-CY7 | BD Biosciences | 562698 |
|  | CD45 | APC-CY7 | BD Biosciences | 560178 |
|  | CD61 | FITC | BD Biosciences | 557291 |
|  | CD62p | PE | BD Biosciences | 555524 |
|  | LIVE/DEAD | FVS 700 | BD Biosciences | 564997 |
| **T cells** | HLA-DR | FITC | BD Biosciences | 555811 |
|  | CD3 | BV510 | BD Biosciences | 563109 |
|  | CD4 | BV421 | BD Biosciences | 566907 |
|  | CD8 | BV711 | BD Biosciences | 563677 |
|  | CD38 | APC | BD Biosciences | 555462 |
|  | CD45 | APC-CY7 | BD Biosciences | 560178 |
|  | CD45RA | PE-CY7 | BD Biosciences | 649457 |
|  | CCR7 | PE | BD Biosciences | 560765 |
|  | LIVE/DEAD | FVS 700 | BD Biosciences | 564997 |
| **B cells** | IgD | FITC | BD Biosciences | 555778 |
|  | CD3 | BV510 | BD Biosciences | 563109 |
|  | CD19 | PC5.5 | BD Biosciences | 566396 |
|  | CD20 | BV421 | BD Biosciences | 562873 |
|  | CD24 | PE | BD Biosciences | 555428 |
|  | CD27 | PE-CY7 | BD Biosciences | 560609 |
|  | CD38 | APC | BD Biosciences | 555462 |
|  | CD45 | APC-CY7 | BD Biosciences | 560178 |
|  | LIVE/DEAD | FVS 700 | BD Biosciences | 564997 |
| **Th1/2/17** | HLA-DR | FITC | BD Biosciences | 555811 |
|  | CD3 | BV510 | BD Biosciences | 563109 |
|  | CD4 | BV421 | BD Biosciences | 566907 |
|  | CD8 | BV711 | BD Biosciences | 563677 |
|  | CD38 | APC | BD Biosciences | 555462 |
|  | CD45 | APC-CY7 | BD Biosciences | 560178 |
|  | CD183 | PE | BD Biosciences | 557185 |
|  | CD196 | PE-CY7 | BD Biosciences | 560620 |
|  | LIVE/DEAD | FVS 700 | BD Biosciences | 564997 |
| **Treg** | HLA-DR | FITC | BD Biosciences | 555811 |
|  | CD3 | BV510 | BD Biosciences | 563109 |
|  | CD4 | BV421 | BD Biosciences | 566907 |
|  | CD25 | PC5.5 | BD Biosciences | 566447 |
|  | CD45 | APC-CY7 | BD Biosciences | 560178 |
|  | CD45RO | PE | BD Biosciences | 555493 |
|  | CD127 | APC | BD Biosciences | 558598 |
|  | CD194 | PE-CY7 | BD Biosciences | 557864 |
|  | LIVE/DEAD | FVS 700 | BD Biosciences | 564997 |
| **DC/mono/NK** | HLA-DR | FITC | BD Biosciences | 555811 |
|  | CD3 | BV510 | BD Biosciences | 563109 |
|  | CD11c | BV786 | BD Biosciences | 740966 |
|  | CD14 | PE-CY7 | BD Biosciences | 562698 |
|  | CD16 | BV605 | BD Biosciences | 740436 |
|  | CD19 | PC5.5 | BD Biosciences | 566396 |
|  | CD20 | BV421 | BD Biosciences | 562873 |
|  | CD45 | APC-CY7 | BD Biosciences | 560178 |
|  | CD56 | PE | BD Biosciences | 555516 |
|  | CD123 | APC | BD Biosciences | 560087 |
|  | LIVE/DEAD | FVS 700 | BD Biosciences | 564997 |

|  | **Retrospective study** | | **P value** |
| --- | --- | --- | --- |
|  | **LPE group (n=32)** | **PE group (n=25)** |  |
| **Female sex (n, %)** | 29 (90.6%) | 22 (88.0%) | P>0.05 |
| **Age (y; mean, SD)** | 46.91 (16.20) | 43.24 (17.43) | P>0.05 |
| **Age at onset (y; mean, SD)** | 43.97 (16.17) | 41.33 (17.21) | P>0.05 |
| **Course of disease (m; median, IQR)** | 15.50 (58.00) | 13.00 (61.00) | P>0.05 |
| **ARR (n; median, IQR)** | 0.62 (1.85) | 0.55 (0.60) | P>0.05 |

**Table 2 The patient characteristics in retrospective study**

**Table 3** **Complete blood count changes in the retrospective cohort**

| **Blood cells** | **Before LPE** | **After LPE** | **P value** |
| --- | --- | --- | --- |
| Erythrocytes (10^12/L) | 4.27±0.67* | 3.98±0.73 | P<0.001 |
| Hemoglobin (g/L) | 117.26±13.95 | 110.78±14.42 | P=0.002 |
| Platelets (10^9/L) | 231.74±69.46 | 185.61±62.73 | P<0.001 |
| Leukocytes (10^9/L) | 7.05±2.57 | 7.42±2.27 | P>0.05 |
| Neutrophils (10^9/L) | 5.84±2.45 | 6.03±1.85 | P>0.05 |
| Lymphocytes (10^9/L) | 1.39±0.43 | 1.50±0.59 | P>0.05 |
| Monocytes (10^9/L) | 0.58±0.17 | 0.60±0.26 | P>0.05 |
| *Data format: mean ± standard deviation | | | |

**Table 4 Changes in complete blood count and inflammatory parameters after LPE treatment in the prospective cohort**

| **Test Item** | **Before LPE1** | **After LPE1** | **Before LPE2** | **After LPE2** | **P value^#^** |
| --- | --- | --- | --- | --- | --- |
| Leukocytes (10^9/L) | 8.40 (5.05)***** | 9.10 (5.65) | 11.70 (5.55) | 9.60 (5.70) | P=0.024 |
| Erythrocytes (10^12/L) | 4.05 (0.76) | 3.94 (0.70) | 3.98 (0.64) | 3.76 (0.63) | P<0.001 |
| Neutrophils (10^9/L) | 6.80 (5.25) | 6.50 (5.25) | 9.70 (4.10) | 7.60 (5.50) | P=0.037 |
| Lymphocytes (10^9/L) | 1.30 (0.95) | 1.40 (1.00) | 1.50 (0.85) | 1.50 (1.10) | P>0.05 |
| Monocytes (10^9/L) | 0.50 (0.40) | 0.60 (0.45) | 0.70 (0.50) | 0.70 (0.35) | P=0.001 |
| Platelets (10^9/L) | 202.00 (60.00) | 137.00 (45.00) | 170.00 (44.50) | 125.00 (42.00) | P<0.001 |
| Hemoglobin (g/L) | 120.00 (15.00) | 118.00 (14.00) | 118.00 (14.50) | 114.00 (13.00) | P<0.001 |
| Fibrinogen (g/L) | 2.65 (1.28) | 2.01 (0.84) | / | 1.75 (0.55) | P<0.001 |
| ESR (mm/h) | 14.00 (17.00) | 7.00 (11.00) | / | 5.00 (6.00) | P<0.001 |
| CRP (mg/L) | 1.64 (1.84) | 1.29 (0.76) | / | 1.32 (0.74) | P=0.034 |
| Complement C4 (mg/L) | 173.00 (101.00) | 132.00 (66.00) | / | 113.00 (40.35) | P<0.001 |
| Complement C3 (mg/L) | 920.00 (263.00) | 732.00 (237.00) | / | 679.00 (194.00) | P<0.001 |
| IgG (g/L) | 10.30 (6.40) | 9.49 (3.54) | / | 8.95 (2.81) | P<0.001 |
| IgA (mg/L) | 1730.00 (1105.00) | 1650.00 (780.00) | / | 1670.00 (710.00) | P=0.041 |
| IgM (mg/L) | 1070.00 (1010.00) | 963.00 (737.00) | / | 867.00 (474.00) | P=0.002 |
| IL-1β (pg/ml) | 5.07 (6.75) | / | / | 5.00 (2.23) | P=0.005 |
| TNF-α (pg/ml) | 8.17 (2.89) | / | / | 7.48 (1.85) | P=0.016 |
| IL-6 (pg/ml) | 2.29 (4.49) | / | / | 2.00 (0.185) | P=0.019 |

*Data format: median (IQR); ^#^P-value represents the statistical significance of the difference between values measured before the first LPE and after the second LPE.

**Table 5 The data of 23 cytokines without statistical significance before and after LPE treatments.**

| **Cytokines (pg/ml)** | **Before LPE** | **After LPE** | **P value** |
| --- | --- | --- | --- |
| MIP-1beta | 207.93 (38.49)* | 195.09 (29.66) | P>0.05 |
| IFN-gamma | 1.88 (1.71) | 1.88 (1.48) | P>0.05 |
| IL-1ra | 751.87 (1058.54) | 974.79 (1577.43) | P>0.05 |
| IL-5 | 10.55 (16.08) | 10.55 (23.63) | P>0.05 |
| GM-CSF | 1.47 (1.90) | 1.47 (2.24) | P>0.05 |
| RANTES | 7230.00 (3950.00) | 5646.00 (3273.00) | P>0.05 |
| IL-2 | 1.43 (1.22) | 1.70 (1.34) | P>0.05 |
| Eotaxin | 78.48 (87.51) | 65.32 (82.01) | P>0.05 |
| Basic FGF | 22.64 (8.00) | 22.64 (10.15) | P>0.05 |
| VEGF | 34.60 (82.10) | 58.25 (77.23) | P>0.05 |
| PDGF-BB | 2738.00 (2144.00) | 1956.00 (2402.00) | P>0.05 |
| IP-10 | 503.06 (379.60) | 371.60 (525.95) | P>0.05 |
| IL-13 | 1.05 (0.85) | 0.94 (1.07) | P>0.05 |
| IL-4 | 4.77 (4.54) | 4.31 (3.63) | P>0.05 |
| MCP-1 | 29.49 (60.43) | 28.74 (34.72) | P>0.05 |
| IL-8 | 7.93 (10.86) | 26.57 (63.43) | P>0.05 |
| MIP-1alpha | 3.57 (5.24) | 6.16 (6.94) | P>0.05 |
| IL-10 | 2.61 (3.13) | 2.41 (1.85) | P>0.05 |
| G-CSF | 129.41 (127.28) | 158.49 (158.40) | P>0.05 |
| IL-15 | 89.68 (137.04) | 107.63 (112.82) | P>0.05 |
| IL-7 | 8.60 (6.82) | 8.60 (5.47) | P>0.05 |
| IL-12p70 | 5.64 (4.82) | 5.64 (4.82) | P>0.05 |
| IL-17 | 11.62 (8.12) | 10.91 (8.30) | P>0.05 |

*Data format: median (IQR)
